# Inhibition of Sp1 Functions by Its Sequestration into PML Nuclear Bodies

**DOI:** 10.1371/journal.pone.0094450

**Published:** 2014-04-11

**Authors:** June Li, Wen-Xin Zou, Kun-Sang Chang

**Affiliations:** Department of Translational Molecular Pathology, The University of Texas M. D. Anderson Cancer Center, Houston, Texas, United States of America; Hokkaido University, Japan

## Abstract

Promyelocytic leukemia nuclear bodies (PML NBs) are comprised of PML and a striking variety of its associated proteins. Various cellular functions have been attributed to PML NBs, including the regulation of gene expression. We report here that induced expression of PML recruits Sp1 into PML NBs, leading to the reduction of Sp1 transactivation function. Specifically, Chromatin immunoprecipitation (ChIP) assay demonstrated that induced expression of PML significantly diminishes the amount of Sp1 binding to its target gene promoter, immunofluorescence staining showed dramatic increase in the co-localization between PML and Sp1 upon induction of PML expression, moreover, PML and Sp1 co-fractionated in the core nuclear matrix. Our study further showed that PML promotes SUMOylation of Sp1 in a RING-motif-dependent manner, SUMOylation of Sp1 facilitates physical interaction between Sp1 and PML and recruitment of Sp1 into the PML NBs, the SUMO binding motif of PML was also important for its interaction with Sp1. The results of this study demonstrate a novel mechanism by which PML regulates gene expression through sequestration of the transcription factor into PML NBs.

## Introduction

The nucleus of a cell is compartmentalized into highly organized structural and functional domains, and many of these subnuclear structures are associated with specific cellular functions. The promyelocytic leukemia nuclear bodies (PML NBs), also called PML oncogenic domains (PODs), Kremer (kr) bodies, and nuclear domain 10 (ND10) [1]–[5], are comprised of PML as the essential component and a large number of PML NB-associated proteins. A model of PML NB formation has been proposed, in which PML is first modified by the small ubiquitin-like modifier (SUMO)-1 and then noncovalent binding of PML to SUMOylated PML through the SUMO binding motif (SIM) constitutes the nucleation event for subsequent recruitment of SUMOylated proteins and/or proteins containing SIM to form the PML NBs [6],[7]. Many functionally important proteins have been found to associate with PML in the PML NBs, almost 40% of PML partners have been confirmed to be SUMOylated, suggesting that PML NBs are enriched sites for SUMOylated proteins [8]–[11]. These proteins do not appear to act in a common pathway or to share structural features in common, which may account for the diverse functions of PML NBs, such as DNA damage response and repair, apoptosis, tumor suppression, and transcriptional regulation [12]–[15].

There are numerous reports describing PML as a tumor suppressor, with respect to its function in mediating programmed cell death. PML has been reported to act as a transcriptional activator or a repressor in a target-gene-specific manner. How PML regulate the transcription of target genes is not well understood. Most of the PML proteins are found in the PML NBs, which are tightly bound to the nuclear matrix core, although some PML isoforms are also found in the cytoplasm [16]–[18]. How PML regulates association and dissociation of the NB-associated proteins remains unclear.

Specificity protein 1 (Sp1) was the first transcription factor identified and characterized. Sp1 is widely expressed in all mammalian tissues/cells and plays critical roles in the normal development of tissues/organs. The transcriptional activity of Sp1 is modulated by post-translational modifications that regulate Sp1 protein level, transactivation activity, and DNA binding affinity [19]. The involvement of Sp1 in the development of various cancer types is well known. Several compounds with anti-tumor effects function by inhibiting Sp1 transcriptional activity [20]–[24]. Therefore, investigation on Sp1 holds great promise to provide insight into related carcinogenesis and to develop efficient therapeutic strategies for related cancers.

Our previous study [25] demonstrated that PML and Sp1 are physically and functionally associated in vivo. PML inhibits Sp1-mediated transcriptional activation of the epidermal growth factor receptor (EGFR) gene by interacting with and preventing Sp1 from binding to its promoter. Our current study is to further understand the mechanistic insight of how PML interacts with Sp1 and inhibits Sp1 functions in vivo. We have demonstrated that PML repressed the transactivation function of Sp1 by sequestering Sp1 into the PML NBs. Overexpression of PML promotes the SUMOylation of Sp1. The RING motif of PML and the SUMOylation site of Sp1 are essential for the recruitment of Sp1 into PML NBs. The SUMO binding motif of PML also plays an important role in this process. Together, our study demonstrates a novel mechanism of PML NB regulation of transcriptional silencing by sequestration of transcription factors.

## Material and Methods

### Plasmid constructs

The plasmids pCMV-FLAG-Sp1-HA, pCMV-Flag-Sp1K16R-HA (substitution of lysine 16 with arginine), and pGEX-GST-Sp1 were kindly provided by Dr. Mary L. Spengler [26]. Plasmids PML-K65, PML-K160, and PML-K490 and Ra-HA-SUMO-1 were PML4′s derivations and kindly provided by Dr. Edward T.H. Yeh [27]. Plasmids GFP-PML, GFP-PML-C72A (cysteine 72 was replaced with an alanine), and GFP-PMLΔR (deletion of amino acid 72–75) were PML6 and its derivations, and were generously provided by Dr. M. Dasso [28]. Plasmids PML3 m (lysines 65, 160, and 490 were replaced with an arginine), PMLas (large nonpolar amino acids VVVI, amino acids 526–529, were changed to small nonpolar amino acids AAAS), and PML3mas (combine mutations of PML3 m and PMLas) were derived from PML4 and kindly provided by Dr. Pier Paolo Pandolfi [7]. The plasmid pcDNA3.SUMO-1 was kindly provided by Dr. Charles Sherr [29].

### Cell culture

U2OS, SiHa, and 293T cells, from American Type Culture Collection (Rockvill, MD), were cultured in Dulbecco's modified Eagle's medium (DMEM) supplemented with 10% fetal bovine serum (FBS). Immortalized PML^−/−^ and WT mouse embryonic fibroblast (MEF) cell lines were generated in our lab as described previously [30] and cultured in DMEM containing 15% FBS. Experiments presented were performed using the immortalized cells at passage 17. U2OS stable cell lines including U2OS/pMEP4 (transfected with pMEP4 empty vector) and U2OS/PML4 (transfected with pMEP4/PML4) were established in our lab as described previously [31] and cultured in DMEM supplemented with 10% FBS. Expression of PML4 was induced by the addition of 100 µM ZnSO_4_.

### Chromatin immunoprecipitation

The chromatin immunoprecipitation (ChIP) assay was performed using the ChIP assay kit obtained from Upstate Biotechnology, Inc., according to the manufacturer's instruction. Briefly, U2OS/pMEP4 and U2OS/PML4 stable cells were treated with formaldehyde to crosslink DNA and binding proteins, followed by sonication to shear DNA into small fragments. Immunoprecipitation was carried out overnight at 4°C with anti-Sp1, anti-acetyl histone H3 polyclonal antibody or rabbit IgG. Precipitated DNA was recovered and analyzed by semi-quantitative PCR. The DNA precipitated by using anti-acetyl histone H3 polyclonal antibody was analyzed by semi-quantitative PCR for the GAPDH promoter, which was used to normalize the DNA output of ChIP. The primer sets 5′-CTCGCATTCTCCTCCTCCTCT-3′/5′-CCCGATCAATACTGGACGGAG-3′ and 5′-CACGCGTTCTTTGAAAGCAG-3′/5′-GGCCTTCTGGGAGTAGAGGC-3′ were used for PCR amplification of EGFR and Survivin promoters, respectively. The primer set for the GAPDH promoter was included in the ChIP assay kit as a control. All data shown represent one of three independent experimental results.

### Immunoprecipitation and immunoblotting

Cells were lysed in 1× radioimmunoprecipitation assay (RIPA) buffer, and the soluble fractions (1 mg/ml) were used for immunoprecipitation with PML antibody, anti-Sp1 antibody, or normal rabbit IgG (negative control). The immunocomplexes were collected by the addition of protein A/G plus agarose and then washed three times with RIPA. For immunoblotting, the immunocomplexes were resolved in SDS-PAGE and transferred onto nitrocellulose membranes. Western blot analysis was performed by using anti-Sp1 or anti-PML antibody. ImageJ (http://rsb.info.nih.gov/ij/index.html) was used to quantify the relative density (aka intensity) of bands on western blot. All data shown represent one of three independent experimental results.

### Immunofluorescence staining

SiHa, U2OS, and PML−/− MEFs were grown on cover slides in 6-well plates; cells were fixed in cold 4% paraformaldehyde in PBS for 30 min on ice, washed in PBS, permeabilized in a 1% Triton X-100/0.5% NP40/PBS solution, and blocked in 1% bovine serum albumin (BSA) in PBS. Cells were then treated with cold cytoskeleton stripping buffer. Briefly, cells were placed on ice for 15 min and then treated with cytoskeleton stripping buffer A (10 mM piperazine-N,N′-bis[2-ethanesulfonic acid] [pH 6.8], 100 mM NaCl, 300 mM sucrose, 3 mM MgCl2, 1 mM EGTA, and 0.5% Triton X-100) for 5 min. After three washes with PBS, cells were treated with cytoskeleton stripping buffer B (10 mM Tris-HCl [pH 7.4], 10 mM NaCl, 3 mM MgCl2, 1% Tween-20, and 0.25% sodium deoxycholate) for another 3 min. Cells were then fixed and permeabilized as described above. Incubation with a primary antibody was carried out for 2 h at room temperature. Incubation with a secondary antibody was carried out for 1 h at room temperature, followed by staining of DNA with 4,6-diamidino-2-phenylindole (DAPI) for 1 h. Slides were mounted with Vectashield antifade medium (Vector Laboratories) after three washes with PBS and examined with a Leica DM LB fluorescence microscope. Images were captured with a Leica digital camera (DFC-420) and managed with the Leica Application Suite software. All data shown represent one of three independent experimental results.

### In vitro SUMOylation assay

The in vitro SUMOylation assay was performed using a commercial kit purchased from LAE Biotech (kit # K007) according to the manufacturer's instructions. The GST-Sp1 fusion proteins were purified using glutathione-Sepharose 4B (Pharmacia Corp.) and then used as a substrate in the in vitro assay. A positive control was performed using α-topoisomerase as the substrate. The products were then subjected to Western blot analysis using anti-topoisomerase and anti-Sp1 antibodies to visualize the SUMOylated proteins. In vitro transcription/translation of the expression plasmid pcDNA3/PMLIV is also performed, and the translated PMLIV protein was used as substrate in the assay. After reactions, Western blotting was performed using anti- Sumo-1 (upper panel) or anti-Sp1 (lower panel) antibody to visualize the shifted SUMOylated proteins. All data shown represent one of three independent experimental results.

### Co-fractionation of PML and Sp1 in the nuclear matrix

U2OS/pMEP4 and U2OS/PML4 stable cells treated with 100 µM/ml ZnSO4 for 12 h were harvested and subjected to the nuclear matrix (NM) fractionation procedure, as described previously [17]. Briefly, cell pellets were resuspended in 5 volumes of 0.5% Triton X-100 in PEM (0.1 M PIPES [pH 6.8], 1 mM EGTA, 1 mM MgCl2, and proteinase inhibitors) at 4°C for 5 min. After centrifugation for 2 min at 12,000 g, the supernatant was collected as fraction 1. The cell pellet was resuspended in 5 volumes of buffer (0.1 M sucrose, 10 mM PIPES, 3 mM MgCl2, 1 mM EGTA, proteinase inhibitors, and 100 µg/ml DNase 1) and incubated at 33°C for 1 h. After centrifugation, as described above, the supernatant was collected as fraction 2. The pellet was resuspended in 5 volumes of 0.25 M ammonium sulfate and left on ice for 5 min. After centrifugation, the supernatant was collected as fraction 3. The pellet was resuspended in 5 volumes of 2 M NaCl and incubated on ice for 5 min. After centrifugation, the supernatant was collected as fraction 4. Proteins remaining in the cell pellet represented the core nuclear matrix–associated proteins (fraction 5). The supernatant fractions were concentrated in a Speedvac and adjusted to a final volume of 50 µl before gel electrophoresis and western blot analysis. All data shown represent one of three independent experimental results.

## Results

### Induced expression of PML4 recruits Sp1 into PML NBs

**Figure 1:**
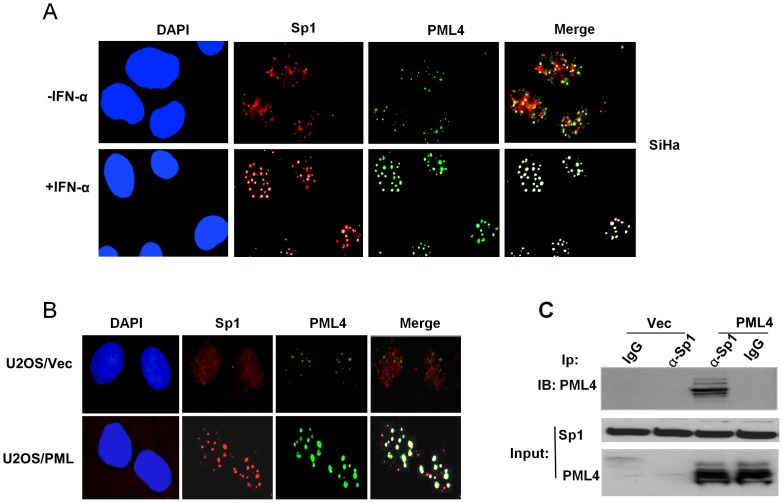
Induced expression of PML recruits Sp1 into nuclear bodies. A. SiHa cells were induced with 2000/ml of α-interferon (IFN-α) for 24 hs, double-color immunofluorescence staining was performed using Sp1 polyclonal and PML monoclonal antibodies. DNA was counterstained with DAPI. B. U2OS/pMEP4 (U2OS/Vec) and U2OS/PML4 (U2OS/PML) cells were treated with 100 µM ZnSO_4_ for 12 hs, and immunofluorescence staining was performed as described above. C. PML4 physically interacts with Sp1. Total protein extracts isolated from U2OS/PML4 and control stable cell lines were subjected to immunoprecipitation using anti-Sp1 antibody with normal rabbit IgG as a negative control. The immunocomplexes were analyzed by Western blotting using PML4-specific antibody.

We have previously reported that PML physically and functionally interacts with Sp1 in vivo and that PML disrupts the binding of Sp1 to its target DNA sequence and inhibits Sp1 transactivation of the target promoter [31]. To further understand the mechanism of PML inhibition of transcriptional activation of Sp1, By double-color immunofluorescence staining, we first examined whether PML and Sp1 co-localize at endogenous levels in SiHa cells in which PML level is relatively higher and is further elevated by interferon induction. The results, as shown in Figure 1A, demonstrated that a substantial amount of Sp1 was being recruited to the PML NBs. A significant increase in Sp1 and PML co-localization was found after treatment of SiHa cells with 2000 U/ml of α-interferon. We also checked Sp1 and PML localization pattern in PML4-inducible expression cell line U2OS/PML4 (U2OS/PML) and the control cell line U2OS/pMEP4 (U2OS/Vec) that were established previously in our laboratory. U2OS/PML cells can be induced to express PML4 by treatment with 100 µM ZnSO_4_ for 12 h. The results of this experiment showed that PML expression level is considerably low in both U2OS/PML cells without ZnSO_4_ treatment and control cells with or without ZnSO_4_ treatment, and the co-localization between Sp1 and PML is undetectable in these cells. However, after ZnSO_4_ induction, Sp1 was recruited to the PML NBs and a significant degree of co-localization between Sp1 and PML was observed in U2OS/PML cells but not in the control cells (Figure 1B). It is worthy noting that Sp1 expression level is not affected by increased PML4 expression. These studies demonstrated that increased PML expression resulted in a dramatic increase in Sp1 recruited to the PML NBs. On the basis of the results of these studies and the results of our previous report [31], we hypothesized that induced PML expression inhibits Sp1 transactivation functions through sequestration of Sp1 into the PML NBs.

We next examined whether PML4 physically interacts with Sp1 in vivo by performing a co-immunoprecipitation assay after induced PML expression in U2OS/PML cells. The results presented in Figure 1C demonstrated that anti-Sp1 antibody but not anti IgG control co-immunoprecipitated the PML4 protein, confirming that the two proteins do physically interact in vivo. This interaction was also confirmed in SiHa cells with or without INFα treatment by similar co-immunoprecipitation assay (Data not shown).

### Overexpression of PML decreases the transcriptional activity of Sp1

**Figure 2:**
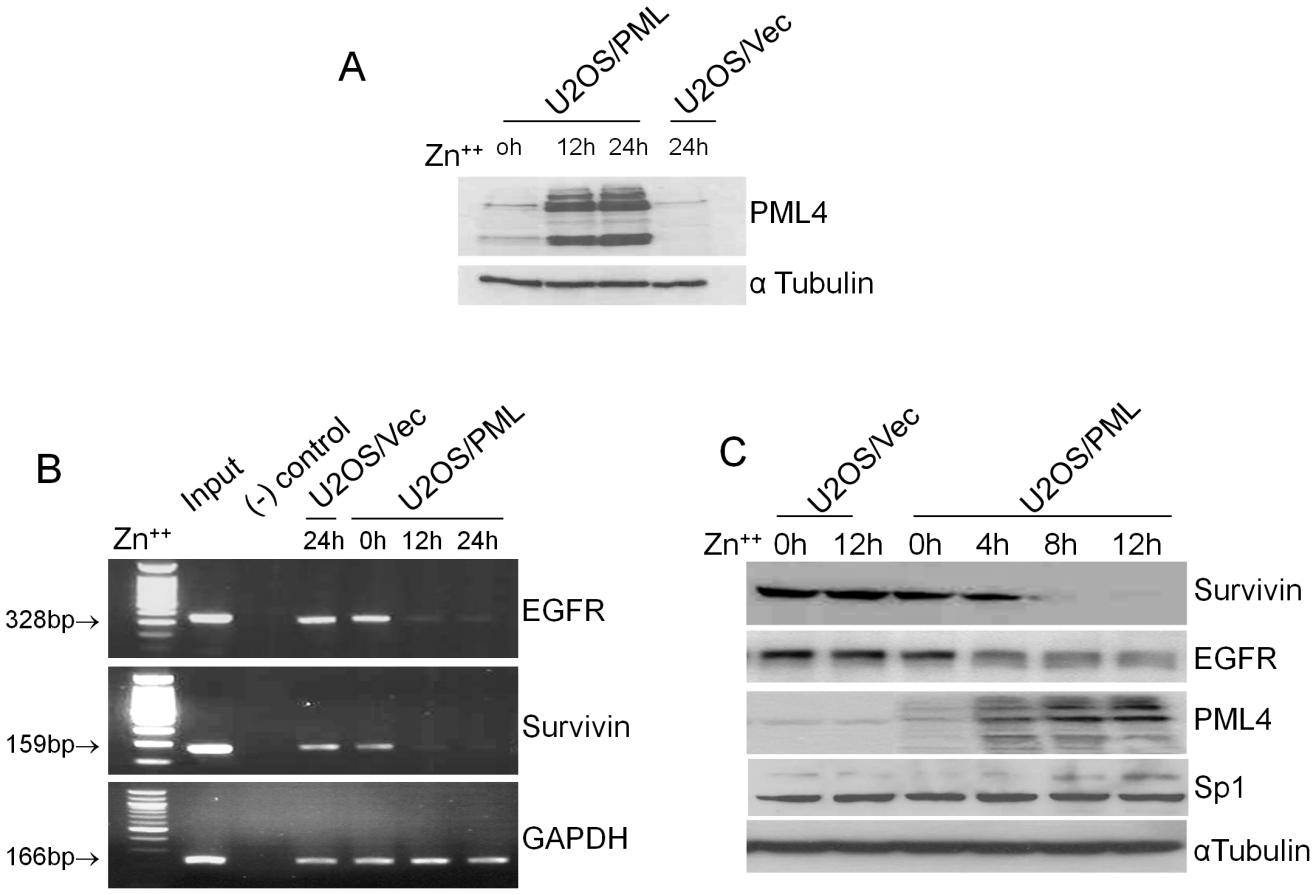
Induced PML expression decreased the transcriptional activity of Sp1 target genes. A. Western blot analysis of the expression of PML4 in U2OS/Vec and U2OS/PML cells after treatment with ZnSO_4_ for 12hs and 24hs. The same filter was re-probed with α-tubulin antibody to serve as a loading control. B. ChIP assay was performed using the stable cell lines U2OS/Vec and U2OS/PML after treatment with ZnSO_4_ for 12 hs. Semi-quantitative PCR was performed using the primer sets for EGFR and Survivin promoters to amplify the chromatin-associated DNA fragments immunoprecipitated by anti-Sp1. A negative control using rabbit IgG was also included. PCR amplification of total chromatin before immunoprecipitation with the same primer sets was considered as “Input”. For semi-quantitative PCR, the DNA samples precipitated by anti-acetyl histone H3 polyclonal antibody were used for PCR amplification of the GAPDH promoter and to normalize the DNA output of each ChIP sample. C. The effects of induced PML4 expression on the expression of EGFR and Survivin by Western blot analysis. U2OS stable cell lines were treated with 100 µM ZnSO_4_, and total proteins were isolated at the indicated time points. Western blotting was performed with EGFR and Survivin antibodies respectively. The same filter was re-probed with α-tubulin antibody to serve as a loading control.

To further investigate how PML4 regulates Sp1 transactivation, we performed ChIP assay using U2OS/PML cells and the control cells to examine whether induced PML4 expression affects Sp1 binding to its target gene promoter in vivo. We chose two putative target genes of Sp1 - EGFR and Survivin for this analysis. The results of this study, presented in Figure 2A and 2B, showed that both EGFR and Survivin promoter sequences were immunoprecipitated by anti-Sp1 antibody but not the IgG control. Moreover, the amount of immunoprecipitated DNA from both EGFR and Survivin promoters are significantly reduced after ZnSO_4_ treatment for 12 and 24 h in U2OS/PML cells compared to in the control cells, as assayed by semi-quantitative PCR. No such reduction was found in the positive control gene GAPDH. Western blot analysis further confirmed that induced PML4 significantly reduced the expression of EGFR and Survivin proteins (Figure 2C). Consistently, expression of both EGFR and Survivin are increased in PML-/- MEFs, as compared to wild type MEFs (Data not shown). The results of this study strongly indicate that induced PML4 expression inhibits transactivation of Sp1 through reducing Sp1 binding to its target gene promoter in vivo.

### PML and Sp1 are co-fractionated with the core nuclear matrix

**Figure 3:**
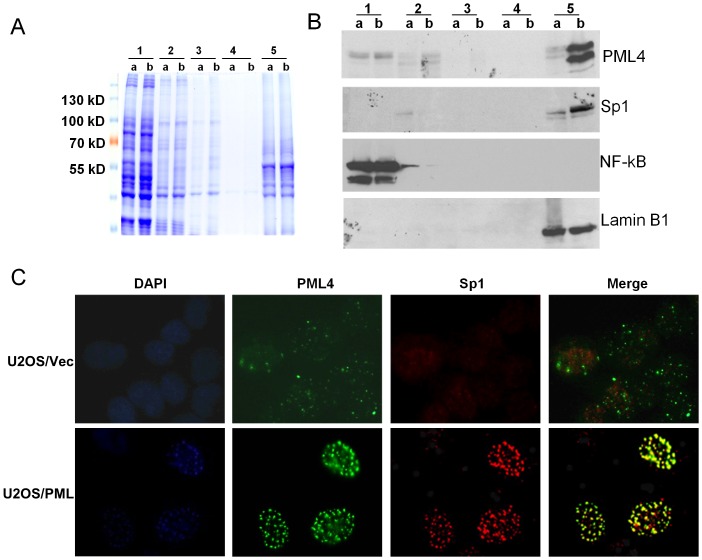
PML and Sp1 are tightly associated in the core nuclear matrix. U2OS/Vec (a) and U2OS/PML (b) cells were treated with 100 µM ZnSO_4_ for 12 h to induce PML4 expression. Numbers 1 though 5 represent different protein fractions isolated from a and b cells according to the procedure described in the material and methods. Different fractions obtained from the NM fractionation were resolved in an acrylamide gel, stained with Coomassie blue (A), and Western blotting analysis with the indicated antibodies (B). Lamin B1 was used as a marker for nuclear matrix-associated fraction. C. A small portion of the remaining cells from fraction 5 were cytocentrifuged onto microscope slides and then double-color immunofluorescence staining of PML and Sp1 in the nuclear matrix core was performed using PML monoclonal and Sp1 polyclonal antibodies.

Our previous study demonstrated that PML is an NM-associated protein [17]. To further confirm the results shown above, U2OS/Vec and U2OS/PML cells treated with ZnSO_4_ for 12 h were subjected to the NM fractionation procedure, as described in Materials and Methods. Different fractions were resolved in an acrylamide gel, stained with Coomassie blue (Figure 3A), and also analyzed by western blotting. The results of this analysis, shown in Figure 3B, are consistent with our previous finding that most PML proteins were recovered from the NM core (fraction 5) in both U2OS/PML cells and U2OS/Vec cells. We found that Sp1 and PML proteins were co-fractionated in the core NM fraction in U2OS/PML cells but not in the control cells. It is also interesting to note that some degree of Sp1 was stripped off the nuclear fraction into the supernatant after DNase 1 treatment (fraction 2) in the control U2OS/Vec cells, but no detectable Sp1 protein could be found in the same fraction in U2OS/PML cells. This observation suggests that in PML4-overexpressing cells, Sp1 was recruited to the PML NBs and tightly associated with the NM. Double-color immunofluorescence staining of the PML and Sp1 proteins in the cells from fraction 5, as presented in Figure 3C, confirmed that Sp1 co-localized with PML NBs in the NM in PML-overexpressed U2OS/PML cells but not in the control U2OS/Vec cells. These results, together with our previous report [32], revealed that PML recruits Sp1 to the PML NBs and sequesters Sp1 transcriptional functions by limiting its accessibility to the target gene promoters.

### Induced expression of PML promotes Sp1 SUMOylation in vivo and in vitro

**Figure 4:**
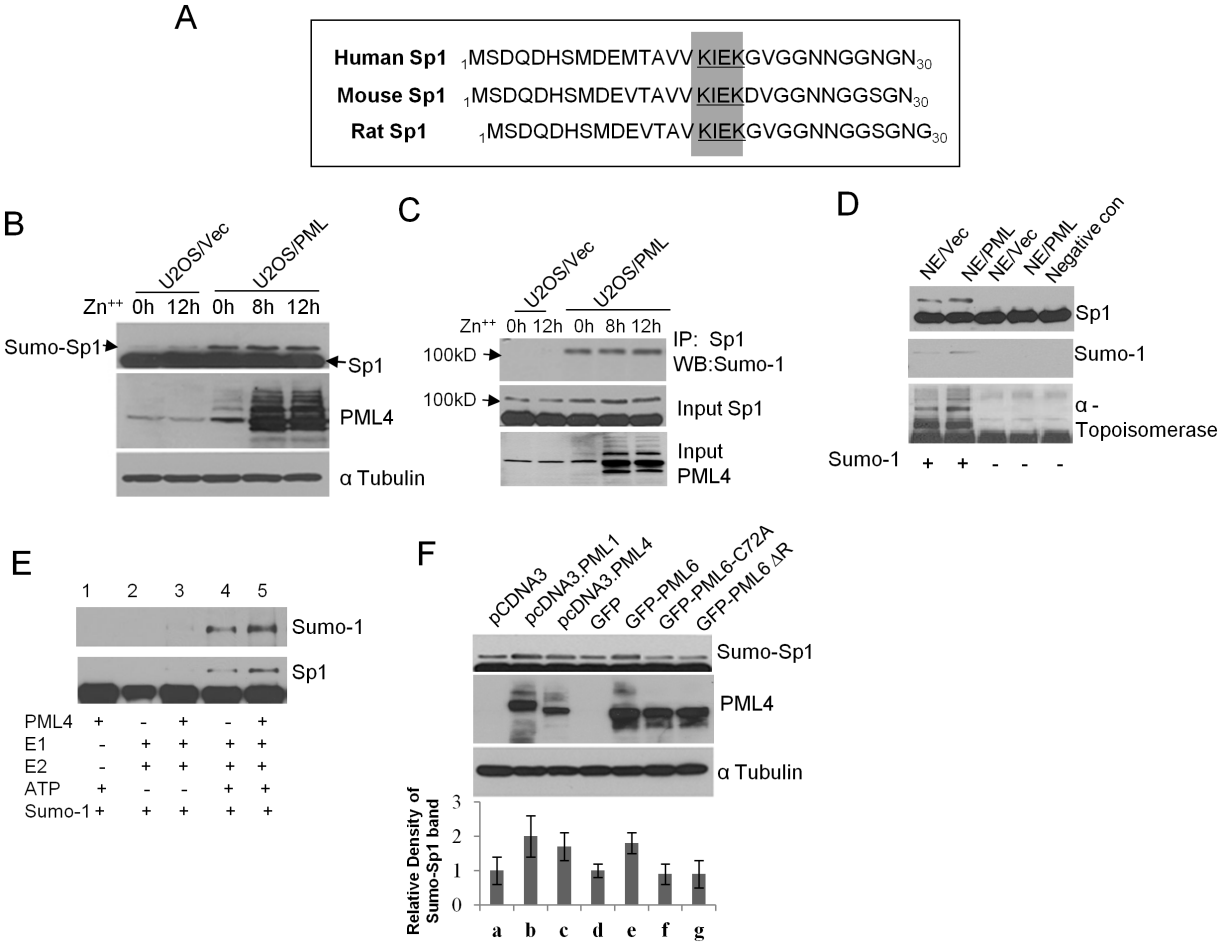
PML promotes Sp1 SUMOylation in vivo and in vitro. A. The consensus sites of SUMOylation in Sp1 in different species are highlighted. B. PML induced SUMOylation of Sp1 in vivo. Total proteins were isolated from U2OS/Vec and U2OS/PML cells induced with 100 µM ZnSO_4_ for 0, 8, and 12 h respectively. Western blot analysis was performed using antibodies against Sp1 and PML. α-tubulin serves as loading control. C. PML induced SUMOylation of Sp1. Immunoprecipitation was performed using Sp1 antibody followed by western blotting with SUMO-1 antibody. D. PML promotes SUMOylation of Sp1 in vitro. In vitro SUMOylation assay was performed using a commercial assay kit from LAE Biotech. The NE (nuclear proteins extract) isolated from U2OS/Vec and U2OS/PML were used in the in vitro assay system. GST-Sp1 or α-topoisomerase (positive control provided with the kit) was used as a substrate. Reaction mixture with NE isolated from U2OS/Vec without SUMO-1 was used as a negative control. Western blotting analysis of final products was performed using anti-Sp1, anti-Sumo-1 and anti- respectively. E. PML promotes SUMOylation of Sp1 in vitro. Same commercial assay kit from LAE Biotech was used. In vitro transcription/translation of the expression plasmid pcDNA3/PMLIV is performed, and the translated PMLIV protein was used as substrate in the assay. Western blotting was performed to check final products using anti-Sumo-1 or anti-Sp1 antibody to visualize the shifted SUMOylated proteins. F. The RING motif is important for PML's activity for promoting Sp1 SUMOylation. Cells (293T) were transfected with pcDNA3 (a), pcDNA3 PML1(b), pcDNA3 PML4 (c), GFP (d), GFP-PML6 (e), GFP-PML6 ΔR (f), and GFP-PML6 C72A (g) respectively. Total proteins were isolated from each transfected cells and western blot analysis was performed using Sp1 and PML antibodies. α Tubulin serves as loading control. All sumo- Sp-1 band density were quantified using ImageJ.

Next, we sought to elucidate how PML recruits Sp1 to the NBs. One major mechanism of recruiting proteins to the PML NBs involves SUMO modifications of the proteins' lysine residues [33],[34]. Sp1 protein consists of a single consensus SUMOylation site, which is conserved between different species (Figure 4A) and is SUMOylated in vitro and in vivo [26]. Furthermore, PML has been shown to stimulate SUMO conjugation in yeast [28]. On the basis of these observations and our new findings presented above, we hypothesized that PML promotes SUMOylation of Sp1 and sequesters Sp1 to the PML NBs.

To test this hypothesis, the stable cell line U2OS/PML and U2OS/PML were induced with ZnSO_4_ to over express the PML4 protein. At various time points, total proteins were isolated and western blotting was performed using SUMO-1 and SUMO-2 antibodies. The results of this study showed a significant increase in the levels of SUMO-modified proteins in PML4-overexpressing cells (Figure S1A). An increase in SUMO-modified proteins was also observed when SiHa cells were treated with interferon to induce PML over expression (Figure S1B). Western blot analysis with anti-Sp1 antibody revealed a significantly higher level of SUMOylated Sp1 in U2OS/PML cells than in the control cell line U2OS/Vec, after ZnSO4 induction (Figure 4B). The increase in SUMO-Sp1 was further supported by immunoprecipitation with Sp1 antibody and then western blotting with SUMO-1 antibody (Figure 4C). This finding suggests that induced expression of PML promotes Sp1 SUMOylation in vivo.

We further performed an in vitro SUMOylation assay using the commercial assay kit from LAE Biotech. Western blot analysis of the cytoplasmic extracts (CE) and the nuclear extracts (NE) isolated from U2OS/Vec cells demonstrated the expected nuclear localization of PML, HDAC1, and p53 proteins and the mainly cytoplasmic localization of MEK1 and NF-κB, indication a proper preparation of NE (Figure S1C). Next, we performed an in vitro assay using GST-Sp1 fusion protein as a substrate in the presence of NE isolated from U2OS/PML or U2OS/Vec cells. We found that U2OS/PML cell derived NE significantly increased (5.3-fold) the production of SUMOylated Sp1 compared to U2OS/Vec cell derived NE (Figure 4D, upper panel). Comparable results were obtained when a similar assay was performed using α-topoisomerase as the control substrate (Figure 4D, lower panel). Induced PML4 expression increase production of SUMO-topoisomerase by 4.5-fold. Finally, we performed in vitro transcription/translation of the expression plasmid pcDNA3/PMLIV, and the translated PMLIV protein was used in the in vitro SUMOylation assay described above. The result of this study consistently demonstrated that the in vitro translated PML protein indeed promotes SUMOylation of Sp1 (Figure 4E). Together, the results presented above demonstrate that PML4 promotes Sp1 SUMOylation, suggesting that PML4 may have E3-ligase like activity.

### The RING motif of PML4 is important for the recruitment of Sp1 into PML NBs

All PML isoforms share the same N-terminal rogion containing the RING motif that is known to be critical for the E3 ligase like activity of PML in yeast system [28]. We, therefore, investigated whether the RING motif of PML is important for Sp1 SUMOylation in mammalian cells. PML 1, PML4, PML6, and the RING domain mutants PML6 VIΔR (change cysteine 72 to alanine)and PML6 VIC72A (aa 72-75 deletion) were co tranfected respectively with SP1 into 293T cells. Western blotting results, as presented in Figure 4F, demonstrate that PML I, PML4, and PML6 promoted SUMOylation of Sp1. However, neither increase in Sp1 SUMOylation nor increase in SUMOylated proteins (Figure S1D) was found in cells transfected with the RING domain mutantsThis finding suggests that SUMOylation promoting activity of PML is not isoform specific, and the RING motif of PML is critical for the SUMOylation promoting activity of PML. Giving that PML6 and its RING domain mutant plasmids have been successfully used in Dr. Dasso group's work, we got them from Dr. Dasso and simply used them in our study because it is reasonable to consider PML6 as a representatitive of all PML isoforms in the functional study of the common RING motif of PML.

**Figure 5:**
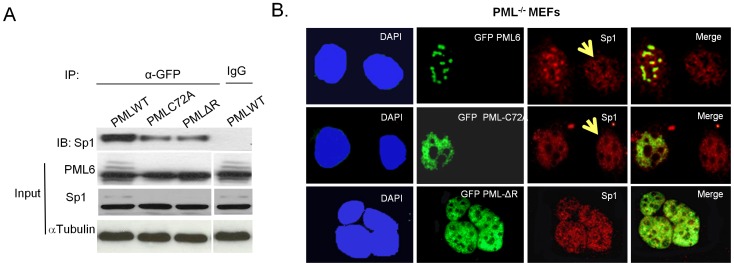
RING domain of PML is essential for the recruitment of Sp1 into the PML NBs. A. Immortalized PML^−/−^ MEFs established as described in our previous report [38] were transfected with GFP -PML4 (PML WT), RING-domain mutants GFP-PML-C72A and GFP-PML-ΔR, respectively. Cells were harvested 24 h post-transfection, and immunoprecipitation was performed using anti-GFP antibody or IgG (negative control) and western blotting was performed with anti-Sp1 antibody. 10% of protein used for immunoprecipitation was used for inputs detection by western blotting B.Double-color immunofluorescence staining was performed using Sp1 polyclonal and PML monoclonal antibodies in PML^−/−^ MEFs transfected with the respective plasmids. DNA contents were counterstained with DAPI.

Next, we would ask whether RING motif of PML4is required for the recruitment of Sp1 to the PML NBs. To the end, GFP-PML6, GFP-PML6-C72A and GFP-PML6 ΔR plasmids were transfected into PML^−/−^ MEFs seperately, immunoprecipitation and western blotting were performed with anti-PML and anti-Sp1 antibodies respectively. We found that the RING-motif mutants immunoprecipitated significantly less Sp1, as shown in Figure 5A and 5B. Immunofluorescence staining revealed that transfected PML forms NBs and co localizes with Sp1 that is recruited in the NBs, while PML-C72A and PML-ΔR display a predominantly nuclear diffuse staining pattern, so does Sp1 in the same cells (Figure 5C). Interestingly, Sp1 in PML^−/−^ MEFs without transfection of PML also show nuclear diffuse staining pattern, as arrow pointed in Figure 5C. Collectively, these results suggest that the RING domain of PML play an important role for the recruitment of Sp1 into PML NBs.

### SUMO binding motif (SIM) of PML4 affects interaction with Sp1

**Figure 6:**
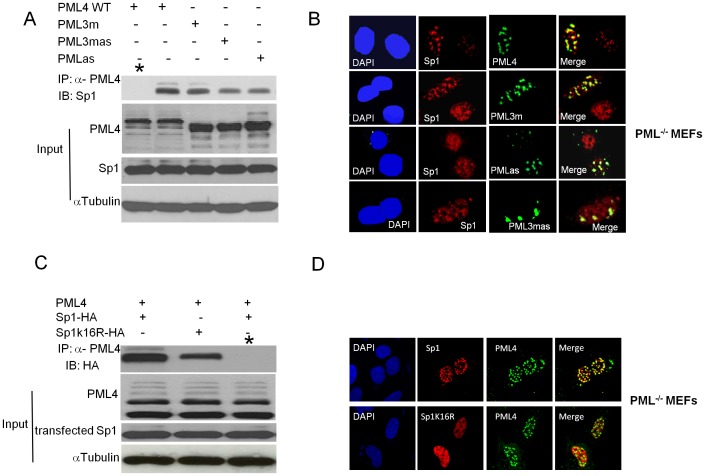
SIM plays a role in the recruitment of Sp1 into the PML NBs. A. Wild-type PML, PML3 m, PMLas, and PML3mas expression plasmids were transfected into PML^−/−^ MEFs separately. Total proteins were isolated from different plasmid transfected cells and co-immunoprecipitations using anti-PML antibody were performed, * in the left panel represents a negative control with anti IgG, and western blotting was carried out with anti-Sp1 antibody. Western blotting on 10% of protein used for immunoprecipitation was performed for inputs detection. B. Double-color immunofluorescence staining with Sp1 polyclonal and PML monoclonal antibodies was performed in PML^−/−^ MEFs transfected with different plasmids described in A. DNA were counterstained with DAPI. C. The Sp1 SUMOylation site is essential for physical interaction with PML. PML^−/−^ MEFs were co-transfected by PMLIV with pCMV-FLAG-Sp1-HA or pCMV-FLAG-Sp1K16R-HA. Immunoprecipitation was performed with anti-PML antibody, and Western blotting was performed with anti-HA antibody. * in the right panel represents a negative control with anti IgG, Western blotting on 10% of protein used for immunoprecipitation was performed for inputs detection. D. Double-color immunofluorescence staining with HA antibody and PML monoclonal antibodies was performed in the transfected cells as described in C, followed by confocal microscopic analysis.

The SIM motif of PML is important for protein-protein interaction and plays a critical role in the organization of PML NBs through interactions with SUMOylated PML and other SUMOylated proteins [6],[7]. We next sought to examine whether the SIM motif of PML affects the recruitment of Sp1 into PML NBs. To this end, we transfected PML4 and its derivation, including PML3m (lysines at amino acids 65, 160, and 490 were replaced with an arginine), SIM mutant PMLas (amino acids VVVI [526–529] were replaced with AAAS), and PML3mas (combined mutations of PMLas and PML3 m), respectively into PML^−/−^ MEFs. Immunoprecipitation was performed using anti-PML antibody, and western blotting was performed with anti-Sp1 antibody. The results of this study showed that PMLIV and PML3 m coimmunoprecipitated comparable amounts of Sp1. However, the level of precipitated Sp1 was moderately reduced in PMLas and PML3mas-transfected samples (Figure 6A), suggesting that SIM is important for physical interaction between PML and Sp1. Immunofluorescence staining results showed that PML4 and all PML mutants co-localized with Sp1; however, a higher degree of Sp1 co-localization was found with PML4 and PML3 m than with PMLas and PML3mas (Figure 6B). Taken together, the results suggest that SIM, but not the SUMOylation sites of PML4, affects the interaction between Sp1 and PML.

### Sp1 SUMOylation site is important for the recruitment of Sp1 into PML NBs

To investigate whether Sp1 SUMOylation is important for its association with PML NBs, we co-transfected the expression plasmids pCMV-FLAG-Sp1-HA and pCMV-FLAG-Sp1K16R-HA (substitution of lysine 16 with an arginine) respectively with PMLIV into PML-/- MEFs cells. After 24 h, the transfected cells were lysed and immunoprecipitation was performed using anti-PML polyclonal antibody and IgG control, and western blotting was carried out using an anti-HA monoclonal antibody. It showed that the amount of Sp1 protein precipitated is significantly reduced in Sp1K16R transfected cells compared to in WT Sp1 transfected cells, while no Sp1 protein was precipitated by IgG (Figure 6C). We also performed double-color immunofluorescence staining by anti HA and anti PML4 antibodies, confocal microscopic analysis presented in Figure 6D clearly demonstrated that colocalization between Sp1K16R-HA and PMLIV is much less compared to that between Sp1 Sp1-HA and PMLIV. Together, this study demonstrated that SUMO modification of Sp1 on amino acid 16 is important for PML and Sp1 association in vivo and recruitment of Sp1 to the PML NBs.

## Discussion

It is now clear that PML and PML NBs control gene expression via regulation of the transcriptional activity of transcription factors. Many of the biological functions of PML depend on its ability to regulate gene expression. Our previous study showed that PML inhibits Sp1-mediated transcriptional activation of the promoter of EGF receptor gene [25]. We speculated that PML-mediated transcriptional repression is not limited to the EGFR promoter but include a broad base of Sp1 target genes. In the present study, we demonstrated, by using ChIP assay and Western blotting, that induced PML expression repressed the transcriptional activity of Sp1 on both Survivin and EGFR genes. This result is consistent with our previous reports [23],[30]. Our study further showed that Sp1, a ubiquitously expressed transcription factor that normally displays a nuclear diffuse cellular localization pattern, was recruited to the PML NBs upon induction of PML over expression by either interferon in SiHa cells or ZnSO4 treatment in a U2OS/PML stable cell line. These observations support the hypothesis that PML regulates Sp1-mediated gene expression by sequestering Sp1 and limiting its accessibility to the target gene promoters. This study also suggests that PML NBs act as a site of temporal storage for Sp1.

PML-NBs could act as ‘modification factories’ with concentrated modification enzymes, where PML brings certain molecules in for post-translational modifications including SUMOylation [35],[36] or targeting cellular proteins to PML NBs requires covalent attachment of SUMO to a consensus lysine residue [37],[38]. Results presented in our study suggest that PML promotes SUMOylation of Sp1, leading to the re-localization of SUMOylated Sp1 into PML NBs. In vitro assay showed that PML overexpression also promotes SUMOylation of the control substrate α-topoisomerase, besides Sp1, suggesting that PML-promoting SUMOylation activity is not Sp1 specific. Although, the activity of PML to promote SUMO conjugation in yeast has been reported [28], the results in the present study are the first to demonstrate the SUMOylation-promoting activity of PML in mammalian cells in a RING motif-dependent manner. Together, our study here demonstrates that induced expression of PML enhances SUMOylation of Sp1 and sequesters sumoylated Sp1 into the PML NBs.

Our results presented in Figure 1 showed that induced expression of PML leads to a substantial increase in the Sp1 recruitment to the PML NBs (almost 100% colocalization between the two proteins). However, the results presented in Figure 4 showed only a small portion of Sp1 is in the SUMOylated form. This discrepancy can be explained by a process of rapid de-SUMOylation during cell lysate preparations, western blotting, or co-immunoprecipitation, presumably by isopeptidases.

Although the over expression of PML was used for the most of the studies. We believe that PML has the same effects on Sp1 at the physiological level. The concept is that more PML have stronger effects. SUMOylation of transcription factors has been reported to have a range of different effects on their transcriptional activity. For example, SUMO modification of Tcf-4 [39] or the heat shock factor HSF1 [40] has been shown to increase their transactivation capacity. However, it is becoming clear that in the vast majority of cases described to date, conjugation of SUMO suppresses the activity of transcriptional factors [41]-[43]. Our study reveals an important molecular mechanism by which conjugated SUMO elicits transcriptional suppression.

It has also been well documented that overexpression of Survivin and EGFR contributes to tumorigenesis in many different types of cancer. For example, Survivin, a suppressor of apoptosis, is overexpressed in most human neoplasms. Increased expression of Survivin is believed to increase cancer cell survival, an important mechanism of cell transformation. Overexpression of Survivin also has prognostic relevance for some tumors and appears to be involved in tumor cell resistance to anticancer drugs and ionizing radiation. Inactivation of either Survivin or EGFR have been considered as new therapeutic approaches for anticancer interventions [44],[45]. We expect that increased Sp1-mediated gene expression occurs as a result of PML deficiency, a consequence that might contribute to the development of various cancers, which was supported by a large-scale study reported by Gurrieri et al. showing that PML deficiency was a frequent event in tumors of many different histological origins [46]. It is our speculation that PML deficiency might serve as a marker for cancer prediction, diagnosis, or targeting PML might become an efficient strategy for certain cancers treatment in the future.

## Supporting Information

Figure S1 **Overexpression of PML promotes SUMOylation in vivo.** A. U2OS/PML stable cells and U2OS/pMEP4 control cells were treated with 100 µM ZnSO_4_ for 0, 4, 8, and 12 h, cells lysates were prepared, and Western blot analysis was performed using antibodies specific for PML4, SUMO-1, SUMO-2/3, and α-tubulin respectively. B. SiHa cells were treated with IFN-α (1000 U) for 24 h, cell lysates were prepared, and Western blot analysis was performed by using antibodies specific for PML, SUMO-1, SUMO-2/3 and α-tubulin respectively. C. Cytoplasmic fraction (CE) and nuclear fraction (NE) isolated from U2OS/pMEP4 and U2OS/PML4 cells after 100 µM ZnSO_4_ induction for 12 h were prepared as described in Materials and Methods. Western blot analysis was performed with the antibodies shown on the side of each panel. D. 293T cells were transiently transfected with GFP-PML6 and RING-domain mutants GFP-PMLC72A and GFP-PMLΔR respectively. At 48 h post-transfection, cell lysates were prepared and Western blot analysis was performed by using antibodies specific for GFP, SUMO-1, and α-tubulin.
(TIF)
